# Cholesterol and Mevalonate: Two Metabolites Involved in Breast Cancer Progression and Drug Resistance through the ERRα Pathway

**DOI:** 10.3390/cells9081819

**Published:** 2020-07-31

**Authors:** Matteo Brindisi, Marco Fiorillo, Luca Frattaruolo, Federica Sotgia, Michael P. Lisanti, Anna Rita Cappello

**Affiliations:** 1 Department of Pharmacy, Health and Nutritional Sciences, University of Calabria, Via P. Bucci, 87036 Rende (CS), Italy; matteo_brindisi@libero.it (M.B.); fiorillo.marco86@gmail.com (M.F.); f.luca90@hotmail.it (L.F.); 2 Translational Medicine, School of Science, Engineering and the Environment (SEE), University of Salford, Greater Manchester M5 4WT, UK

**Keywords:** cholesterol, mevalonate, ERRα, cancer metabolism, CSCs, drug resistance, cancer progression, LDs

## Abstract

Breast cancer is the second greatest cause of cancer-related death in women. Resistance to endocrine treatments or chemotherapy is a limiting drawback. In this context, this work aims to evaluate the effects of cholesterol and mevalonate during tumor progression and their contribution in the onset of resistance to clinical treatments in use today. In this study, we demonstrated that cholesterol and mevalonate treatments were able to activate the estrogen-related receptor alpha (ERRα) pathway, increasing the expression levels of peroxisome proliferator-activated receptor gamma coactivator 1-alpha (PGC-1α), ERbB2/human epithelial receptor (HER2), tumor protein D52 (TPD52), and NOTCH2 proteins in breast cancer cells. The activation of this pathway is shown to be responsible for intense metabolic switching, higher proliferation rates, sustained motility, the propagation of cancer stem-like cells (CSCs), and lipid droplet formation. All of these events are related to greater tumor propagation, aggressiveness, and drug resistance. Furthermore, the activation and expression of proteins induced by the treatment with cholesterol or mevalonate are consistent with those obtained from the MCF-7/TAMr cell line, which is largely used as a breast cancer model of acquired endocrine therapy resistance. Altogether, our data indicate that cholesterol and mevalonate are two metabolites implicated in breast cancer progression, aggressiveness, and drug resistance, through the activation of the ERRα pathway. Our findings enable us to identify the ERRα receptor as a poor prognostic marker in patients with breast carcinoma, suggesting the correlation between cholesterol/mevalonate and ERRα as a new possible target in breast cancer treatment.

## 1. Introduction

Breast cancer is a leading cause of cancer death in women, with almost half a million deaths worldwide per year [1], mainly because of tumor relapse and metastases [2,3]. Furthermore, cancer recurrence is associated with a resistance to common therapies and, for this reason, research aims to continuously identify new therapeutic targets, in order to overcome this phenomenon. In this context, several research studies have focused on lipid metabolism, storage, and mobilization [4,5]. In recent years, we have witnessed a reassessment of lipid droplets’ role, which are lipid-enriched organelles mainly consisting of triacylglycerols and cholesterol esters, delimited by a phospholipidic monolayer. Both the content and nature of their different lipids are very varied, according to the cell type and physio-pathological conditions. Lipid droplets, initially considered inert cytoplasmic structures acting as a fat depot, assumed considerable interest due to their involvement in promoting cancer progression and anti-tumor resistance [5]. Recently, the mevalonate pathway, leading to the synthesis of sterols such as cholesterol and isoprenoids, has emerged as a promising therapeutic target [6,7]. In this pathway, 3-hydroxy-3-methyl-glutaryl-CoA reductase (HMGCR) plays a key role; it catalyzes the production of mevalonate from 3-hydroxy-3-methylglutaryl-CoA and is considered the rate-limiting enzyme for the pathway [8]. It is well-known that in many tumors, including those associated with breast cancer, a high expression of HMGCR is related to a higher tumor aggressiveness and poor prognosis [9,10], but it is not very clear which molecule of the pathway is responsible for these processes. Mevalonate is a pivotal intermediate for cholesterol synthesis, as well as a precursor of ubiquinone, which is a main character in mitochondrial bioenergetics [11,12]. It is well-accepted that the mevalonate pathway drives malignant transformation [7,13,14] and since statins are well-established drugs used to lower serum cholesterol in patients, inhibiting the HMGCR [15], several studies have been performed to show that statins exert antitumor effects in human malignancies, including breast cancer [16,17]. However, the role of statins is debatable; indeed, other studies have failed to show any meaningful anti-tumor effect [18]. Preclinical and clinical studies of breast malignancies have demonstrated variable effects of statins, depending on the cell lines and cohorts, respectively [9,19]. Literature data has demonstrated that high serum cholesterol levels are risk factors in the onset of breast cancer and its metabolism is involved in the onset of resistance to therapy with tamoxifen in ER+ breast cancer [20,21]. Moreover, several reports have shown that cholesterol is closely related to the activation of estrogen-related receptor alpha (ERRα), which, for a long time, was considered to be an orphan nuclear receptor [22,23]. ERRα, along with the transcriptional coactivator peroxisome proliferator-activated receptor gamma coactivator 1-alpha (PGC1-α), plays a key role in mitochondrial biogenesis [24,25,26], which is an important cancer feature. Based on this evidence, the goal of this study was to understand whether high levels of cholesterol, through ERRα pathway activation, are able to determine, by themselves, an aggressive and resistant tumor phenotype, or whether another biosynthetic intermediate is required to observe these phenomena. For this reason, in this study, we compared the effects of mevalonate and cholesterol treatment in promoting both proliferation and infiltrating capacity cells in the main breast cancer subtypes, as well as in inducing resistance to pharmacological treatments currently used in clinical practice. Our results show that cancer cells endured clear metabolic switching after mevalonate or cholesterol treatment, increasing in glycolytic and mitochondrial metabolism. They also demonstrate an increased propagation of cancer stem-like cells (CSCs), which are highly dependent on mitochondrial metabolism, as well as an enhancement in the formation of lipid droplets (LDs), which are key players in tumor recurrence, metastases, and drug resistance, respectively [27,28]. This evidence prompted us to speculate that cholesterol and its biosynthetic precursor, mevalonate, can drive aggressiveness and resistance to anti-cancer therapy used for breast cancer treatment, through the activation of ERRα and related pathways.

## 2. Material and Methods

### 2.1. Cell Cultures

All breast cell lines used in this work (MCF7, T47D, MDA-MB-231, and MDA-MB-468) were purchased from the American Culture Collection (ATCC, Manassas, VA, USA). MCF7, T47D, and MDA-MB-231 cells were cultured in DMEM/F12 (Sigma Aldrich, St. Louis, MO, USA) supplemented with 10% Fetal Bovine Serum (FBS, Sigma Aldrich), 2 mM l-glutamine (Gibco, Life Technologies, Waltham, MA, USA), and 1% penicillin/streptomycin (Gibco, Life Technologies). DMEM (High Glucose) (Sigma Aldrich) supplemented with 10% FBS, 2 mM l-glutamine, and 1% penicillin/streptomycin was used to culture MDA-MB-468 cells. Treatments were performed in the above-mentioned media. The concentrations of mevalonate and cholesterol used in this study were chosen based on the results obtained from previous studies [27,29]. All cell lines were cultured at 37 °C in 5% CO2 in a humidified atmosphere, as previously described [30,31].

### 2.2. Immunoblot Analysis

For immunoblot analysis, cells were grown to 70–80% confluence and treated with 10 µM cholesterol, 1 mM mevalolactone (subsequently defined as mevalonate for simplicity), or EtOH, or co-treated with 5 µM XCT790 for 48 h. Then, cells were harvested and lysed in 200 µL of lysis buffer to produce the total cell lysates, as previously described [32,33]. The same amounts of proteins from total lysates were resolved on SDS-polyacrylamide gel, transferred to a nitrocellulose membrane, and probed with appropriate primary antibodies (Santa Cruz, Biotechnology, Dallas, TX, USA; Abcam, Cambridge, UK). Incubation with the anti-β-actin antibody (Santa Cruz, Biotechnology, Dallas, TX, USA) was used to confirm equal loading and transfer. The complex formed between the antigen and antibody was detected by incubation of the membranes with peroxidase-coupled goat anti-mouse or goat anti-rabbit antibodies and was revealed using the ECL System (Bio-Rad Laboratories, Hercules, CA, USA), as previously described [34]. Then, the bands of interest were quantified by using ImageJ software (Image J, Bethesda, MD, USA).

### 2.3. Cell Cycle Analysis

Cell cycle analysis was performed as previously reported [35]. MCF7, T47D, MDA-MB-231, and MDA-MB-468 cells, seeded in 6-well plates at a density of 1 × 105 cells/well, were treated with EtOH, 10 µM cholesterol, or 1 mM mevalonate for 5 days. After treatment, cells were detached from the wells using trypsin, washed twice with chilled PBS, and centrifuged at 1500 rpm for 5 min. Cells were fixed by re-suspending the pellets in 70% ethanol for 30 min at 4 °C. Then, cells were washed twice with PBS and stained in 3.8 mM sodium citrate, 50 µg/mL propidium iodide [36], 100 µg/mL RNAse, and 0.1% Igepal in PBS for 1 h at 37 °C. Next, samples were subjected to cytofluorimetric analysis using ATTUNE^TM^ (Life Technologies, Grand Island, NY, USA).

### 2.4. Wound-Healing Assay

The wound-healing assay was performed as previously reported [32,37]. Cells were grown to confluence and the monolayers were then scraped using a p200 tip and treated with EtOH, 10 µM cholesterol, or 1 mM mevalonate. Wound closure was monitored for 24 h and cells were then subjected to phase-contrast light microscopy. Pictures were taken at 10× magnification using the EVOS FL Auto 2 microscope (Life Technologies, Grand Island, NY, USA). The rate of wound healing was quantified from the picture using ImageJ software and standard deviations were determined by using GraphPad-Prism 8.3.0 software (GraphPad Inc., San Diego, CA, USA).

### 2.5. Seahorse XFe96 Metabolic Profile Analysis

Extracellular acidification rates (ECAR) and real-time oxygen consumption rates (OCR) for cells treated with mevalonate or cholesterol were assessed using the Seahorse Extracellular Flux (XFe96) analyzer (Seahorse Bioscience, Billerica, MA, USA), in order to evaluate the glycolytic and mitochondrial function, respectively. The analysis was performed as previously described [38]. Briefly, 1 × 10^4^ cells/well were seeded into XFe96-well cell culture plates and incubated overnight in complete medium. Then, cells were treated with mevalonate or cholesterol (1 mM and 10 µM) or co-treated with 5 µM XCT790 for 48 h. Next, cells were washed in pre-warmed XF assay media (or for OCR measurement, XF assay media supplemented with 10 mM glucose, 1 mM Pyruvate, and 2 mM l-glutamine, and adjusted at pH 7.4). Cells were then maintained at 37 °C in a non-CO_2_ incubator for 1 h in 175 µL/well of XF assay media. During the incubation time, we filled up the injection ports in the XFe96 sensor cartridge with 25 µL of 80 mM glucose, 9 µM oligomycin, and 1 M 2-deoxyglucose (for ECAR measurement), or 10 µM oligomycin, 9 µM FCCP, 10 µM rotenone, and 10 µM antimycin A (for OCR measurement), dissolved in the XF assay media. Measurements were normalized by the protein content (SRB assay). Data were analyzed using GraphPad through one-way ANOVA and Student’s t-test calculations and XFe96 software. All experiments were performed in quintuplicate, three times, independently.

### 2.6. Mitochondrial Membrane Potential and Mass Analysis

To measure the mitochondrial membrane potential and mass, cells were stained with the CM-H2TMRos or MitoTracker Deep Red FM probes, respectively (ThermoFisher), as previously described [39]. The accumulation of the CM-H2TMRos probe in mitochondria is dependent upon the membrane potential, while the MitoTracker Deep Red FM is used to label mitochondria, according to the manufacturer’s protocols. Briefly, 1 × 105 cells/well were seeded in 6-well plates and treated with mevalonate, cholesterol, or EtOH for 5 days. After treatment, cells were harvested, rinsed, and incubated in 10 nM MitoTracker Orange solution or 10 nM MitoTracker Deep Red FM in PBS for 30 min at 37 °C. After staining, cells were fixed with 3.7% formaldehyde in PBS at 37 °C for 15 min, rinsed, and resuspended in PBS. Samples were then subjected to cytofluorimetric analysis by using the SONY SH800 Cell Sorter (Sony Corporation, Minato, Tokyo, Japan).

### 2.7. Mammosphere Formation Assay

A single cell suspension was prepared using enzymatic (1× Trypsin-EDTA, Sigma Aldrich) and manual disaggregation (25-gauge needle). Cells were plated at a density of 5000 cells/well in a mammosphere medium (DMEM-F12 w/o red phenol + B27 supplement + 20 ng/mL EGF + penicillin/streptomycin) under non-adherent conditions in 6-well plates pre-coated with 2-hydroxyethylmethacrylate (poly-HEMA, Sigma Aldrich), as previously described [40]. Then, cells were treated with mevalonate or cholesterol at concentrations of 1 mM or 10 µM, respectively. Vehicle alone (EtOH)-treated cells were processed in parallel as a control. Cells were grown for five days and maintained in a humidified incubator at 37 °C. After five days of culture, 3D-spheres > 50 μm were counted using an eye piece (“graticule”), and the percentage of cells plated that formed spheres was calculated, being referred to as the percent mammosphere formation efficiency (MFE).

### 2.8. Immunofluorescence Assay

Breast cancer cells were seeded on coverslips in 6-well plates at a density of 1 × 10^5^ cells/well, and cultured overnight in complete medium. Next, cells were treated for 48 h with mevalonate, cholesterol (1 mM or 10 µM, respectively), or EtOH. After treatment had ended, cells were fixed with ice cold methanol for 20 min at −20 °C, washed three times for 5 min with Tris buffered saline (TBS, Sigma-Aldrich), and incubated for blocking with 10% donkey serum (Sigma-Aldrich) in TBS for 40 min at 37 °C. Then, cells were incubated for 40 min at 37 °C in anti-PLIN monoclonal antibody (Abcam), diluted to 1:200, as previously described [34]. Next, they were washed three times for 5 min with TBS to discard excess primary antibody, incubated for 40 min at 37 °C in anti-rabbit IgG-FITC (Alexa Fluor 488) diluted to 1:500, and subsequently washed three times for 5 min with TBS. DAPI (Sigma-Aldrich, Milan, MI, IT) was also used to stain nuclei. Pictures were taken at 20× magnification using the Olympus BX41 microscope with CSV1.14 software, using a CAMXC-30 for image acquisition.

### 2.9. Sulforhodamine B (SRB) Assay

To assess the cell viability, we used the sulforhodamine B (SRB) assay, as previously described [41]. Briefly, 2 × 10^5^ cells/well were seeded in 48-well plates and cultured overnight to allow the cells to attach. Then, cells were treated with mevalonate or cholesterol in the presence or absence of 4-OH tamoxifen or doxorubicin, for 48 h. At the end of the treatment, cell monolayers were fixed with chilled 10% (wt/vol) trichloroacetic acid for 15 min at 4 °C. Plates were then washed with PBS and allowed to dry. Next, cells were stained with 0.04% (wt/vol) SRB for 30 min, after which the excess dye was removed by washing repeatedly with 1% (vol/vol) acetic acid. The protein-bound dye was dissolved in 10 mM Tris base solution for OD determination at 510 nm using a Varioskan^TM^ LUX microplate reader (ThermoFisher).

### 2.10. Kaplan–Mayer Analysis

The prognostic value of ERRα in breast cancer was evaluated by performing a Kaplan–Meier (K–M) analysis using the updated version of a publicly available microarray database from breast cancer patients (http://kmplot.com/analysis/index.php?p=service&cancer=breast). Relapse-free survival (RFS) was evaluated in a cohort of patients with any breast cancer subtype. Kaplan–Meier correlations were plotted for high (above median, in red) and low (below median, in black) gene expression. Biased array data were excluded from the analysis. Hazard-ratios were calculated, at the best auto-selected cut-off, and *p*-values were calculated using the log-rank test and plotted in R. K–M curves were also generated online using the K–M plotter (as high-resolution TIFF files), as previously reported [42].

### 2.11. Statistical Analysis

Data are presented as mean values ± standard deviation, obtained from ≥3 independent experiments, with ≥3 replicates per experiment, unless otherwise stated. Statistical significance was measured by using the analysis of variance (ANOVA) test. A *p*-value ≤ 0.05 was considered statistically significant. Non-linear regression analysis (GraphPad Prism 8.3.0) was used to generate sigmoidal dose-response curves to calculate the IC_50_ values [43].

## 3. Results

### 3.1. Mevalonate and Cholesterol Activate the ERRα Pathway, Increasing the Levels of Related Proteins

**Figure 1:**
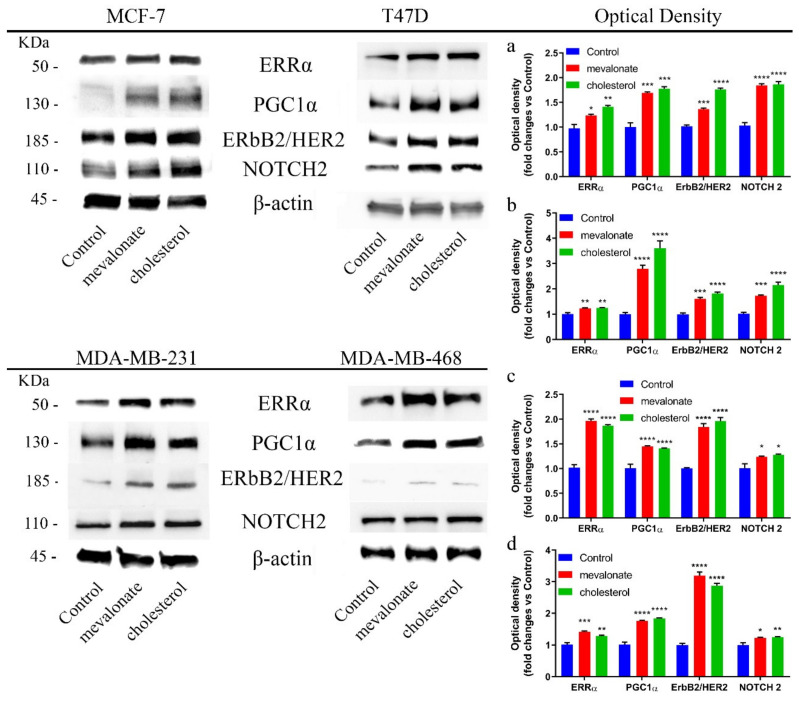
Mevalonate and cholesterol treatment increases the expression levels of estrogen-related receptor alpha (ERRα) pathway-related proteins. Immunoblot analysis of main proteins involved in the ERRα pathway and cell aggressiveness after treatment with 1 mM mevalonate or 10 µM cholesterol for 2 days. Optical density of their expression levels by densitometry obtained in MCF-7 (**a**), T47D (**b**), MDA-MB-231 (**c**), and MDAMB-468 (**d**). Values represent the mean ± SD of three independent experiments. * *p*-value < 0.05; ** *p*-value < 0.01; *** *p*-value < 0.001; **** *p*-value < 0.0001.

In order to evaluate the role of cholesterol and its biosynthetic precursor mevalonate in breast cancer progression, and since the goal of our work was to evaluate whether high cholesterol levels were responsible for greater tumor aggressiveness and resistance phenomena, we supplemented already cholesterol-containing media (10% FBS medium) with the indicated amounts of cholesterol and mevalonate, focusing our attention on the ability of the two metabolites to activate the ERRα pathway in four different breast cancer cell lines. We used cell lines characterized by important differences in the three main receptors conventionally used for breast cancer subtyping: The estrogen receptor (ER), progesterone receptor (PR), and human epithelial receptor (HER 2). In particular, we employed MCF-7 and T47D, which are two breast cancer cell lines characterized by the presence of ERα, and MDA-MB-231 and MDA-MB-468, which are two breast cancer cell lines characterized as triple negative. We found that, in all breast cancer cell lines examined, cholesterol and mevalonate treatment induced a significant increase in ERRα expression levels, as well as in the levels of the related protein PGC-1α—the coactivator needed for the activation of the ERRα pathway—by promoting mitochondrial biogenesis [44] (Figure 1).

**Figure 2:**
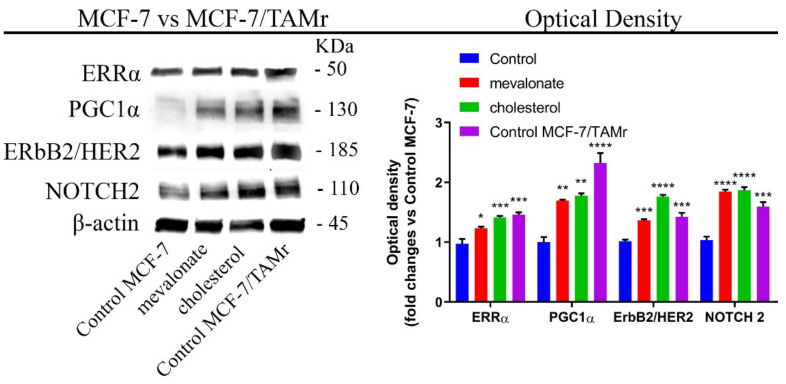
In MCF-7, mevalonate and cholesterol treatment induces similar expression levels of ERRα pathway-related proteins to MCF-7/TAMr cells. Immunoblot analysis of the main proteins involved in the ERRα pathway and cell aggressiveness after treatment with 1 mM mevalonate or 10 µM cholesterol for 2 days. The western blot shown in Figure 2 is the same as the one shown in Figure 1 for MCF7 cells. The optical density of their expression levels was acquired by densitometry obtained in MCF-7 versus MCF-7/TAMr. Values represent the mean ± SD of three independent experiments. * *p*-value < 0.05; ** *p*-value < 0.01; *** *p*-value < 0.001; **** *p*-value < 0.0001.

Additionally, the treatment resulted in an increase of the expression levels of ERbB2/HER2, which is a protein associated with the ERRα pathway with considerable functions in the maintenance of cancer progression, such as the sustenance of proliferation, and significant functions in the metabolism of tumor cells [45,46] (Figure 1). Treatment was also related to increasing levels of NOTCH2, which is a relevant protein in breast cancer propagation and associated with a poor prognosis, indicating the important ability of cholesterol and mevalonate to support the propagation and stemness of breast cancer cells (Figure 1) [47]. The ERRα activation was also evaluated under starvation conditions, and a similar trend was demonstrated (Figure S1). Furthermore, as shown in Figure 2, we compared the expression levels of the above-mentioned proteins in treated MCF-7 cells to those detected in the MCF-7/TAMr cell line, which differs from normal MCF-7 cells by having an acquired resistance to tamoxifen. MCF-7 cells treated with mevalonate or cholesterol exhibited expression levels of both ERRα and related proteins comparable to those of MCF-7/TAMr cells.

**Figure 3:**
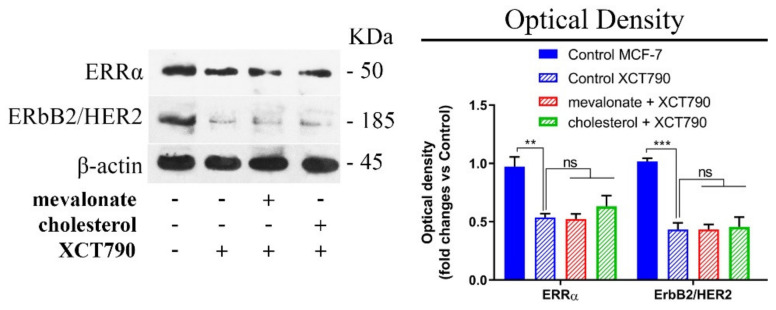
Co-treatment of XCT790 and mevalonate or cholesterol reduces the expression levels of both ERRα and the main correlated proteins in MCF-7 cells. Immunoblot analysis of ERRα and ERbB2/human epithelial receptor (HER2) after co-treatment with 5 µM XCT790, 1 mM mevalonate, or 10 µM cholesterol, for 2 days. Results obtained from densitometry were related to their own control. Values represent the mean ± SD of three independent experiments. ** *p*-value < 0.01; *** *p*-value < 0.001; ns: not significant.

In order to confirm the ability of cholesterol or mevalonate to induce the activation of the ERRα pathway in breast cancer cells, we monitored the ERRα expression levels in cholesterol/mevalonate-treated cells in the presence of XCT790, which is a well-known selective ERRα inhibitor. The presence of the inhibitor in treated MCF-7 cells (representative of ERα cells), as well as in treated MDA-MB-231 (representative of triple negative cells), revealed decreased expression levels of both ERRα and ERbB2 (Figure 3 and Figure S2).

These results, together with the comparable expression levels obtained in treated MCF-7 cells with respect to the MCF-7/TAMr cells, suggest that the ERRα pathway has a role in the acquisition of resistance.

### 3.2. Cholesterol and Its Biosynthetic Precursor, Mevalonate, Lead Cell Cycle Progression

**Figure 4:**
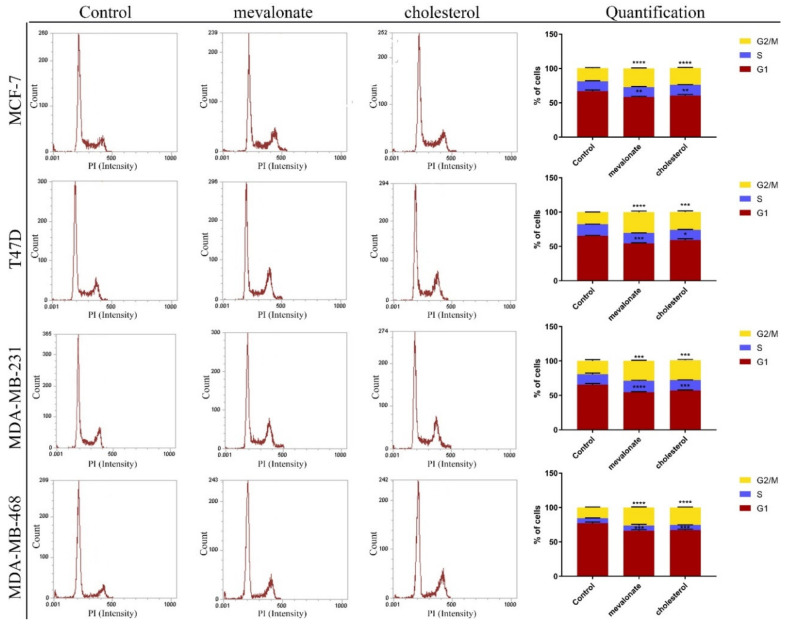
Mevalonate and cholesterol promote cell cycle progression. Cytofluorimetric analysis of MCF7, T47D, MDA-MB-231, and MDA-MB-468 cells treated for 5 days with EtOH (control), 1 mM mevalonate, or 10 µM cholesterol and stained with PI; graphs represent the percentage of the total events in the samples (highlighting the increase in the G2/M phase). Values represent the mean ± SD of three independent experiments. * *p*-value < 0.05; ** *p*-value < 0.01; *** *p*-value < 0.001; **** *p*-value < 0.0001.

Based on the different pathways activated in breast cancer cells by cholesterol or mevalonate and given the pivotal role that they play in promoting tumor proliferation (in particular, ERbB2/HER2 and NOTCH2 signaling), we evaluated the effects of cholesterol or mevalonate treatment on cell cycle progression. The results showed that five days of mevalonate or cholesterol treatment induced a noticeable increase in the G2/M phase (Figure 4), suggesting a greater proliferation rate in all breast cancer cells examined. The proliferative effects were confirmed by cell growth analysis, performed by the SRB assay (Figure S3).

### 3.3. Mevalonate and Cholesterol Improve the Motility of Breast Cancer Cell Lines

**Figure 5:**
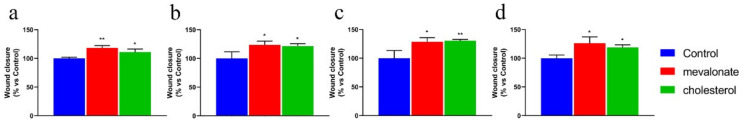
Mevalonate and cholesterol increase breast cancer cell motility. Wound-healing scratch assay in MCF7, T47D, MDA-MB-231, and MDA-MB-468 cells treated for 24 h with EtOH (control), 1 mM mevalonate, or 10 µM cholesterol. Wound closure in MCF-7 (**a**), T47D (**b**), MDA-MB-231 (**c**), and MDAMB-468 (**d**). Values are expressed as the mean ± SD of three independent experiments. * *p*-value < 0.05; ** *p*-value < 0.01.

Next, cell motility, which is a hallmark of cancer aggressiveness [48], was evaluated in all four breast cancer cell lines. For this purpose, cells were seeded, left to reach confluence, and subjected to the wound-healing scratch assay; then, they were treated with mevalonate or cholesterol for 24 h. The pictures were taken using phase-contrast microscopy. Figure 5 and Figures S4–S7 show that, after treatment, cells were able to close the wound faster than each control, highlighting the effects of cholesterol and mevalonate in enhancing cell motility.

### 3.4. Increase of Mitochondrial Activity and Energetic Metabolism in Mevalonate- and Cholesterol-Treated Breast Cancer Cells

**Figure 6:**
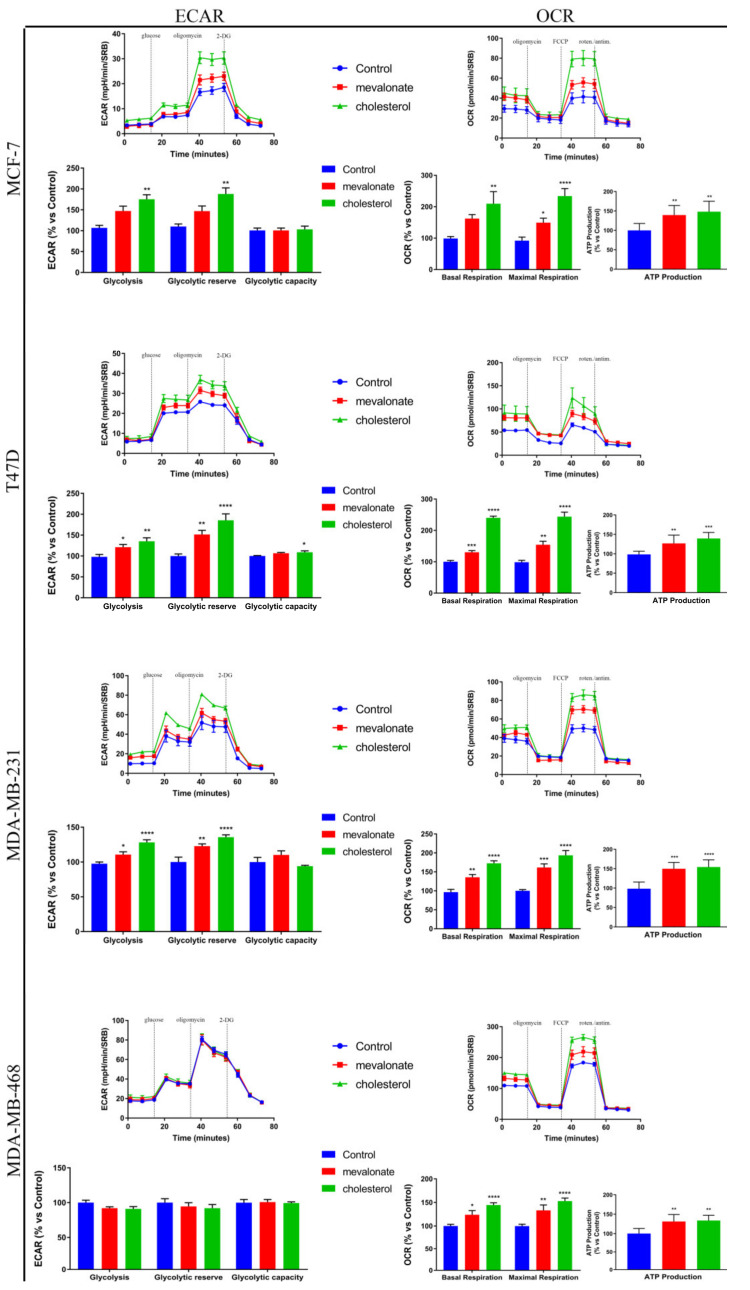
Mevalonate and cholesterol increase metabolic functions in breast cancer cells. A Seahorse XFe96 analyzer was employed to analyze both mitochondrial respiration and the glycolytic function in MCF-7, T47D, MDA-MB-231, and MDA-MB-468 cells treated with mevalonate or cholesterol for 48 h, by monitoring the oxygen consumption rate (OCR) and the extracellular acidification rate (ECAR), respectively, as indicated. OCR representative tracings of breast cancer cells treated with EtOH (control), 1 mM mevalonate, or 10 µM cholesterol and histograms of mitochondrial respiration parameters. ECAR representative tracings of breast cancer cells treated with EtOH (control), 1mM mevalonate, or 10 µM cholesterol and histograms of glycolytic function parameters. Values are expressed as the mean ± SEM of five biological replicates of three independent experiments. * *p*-value < 0.05; ** *p*-value < 0.01; *** *p*-value < 0.001; **** *p*-value < 0.0001.

**Figure 7:**
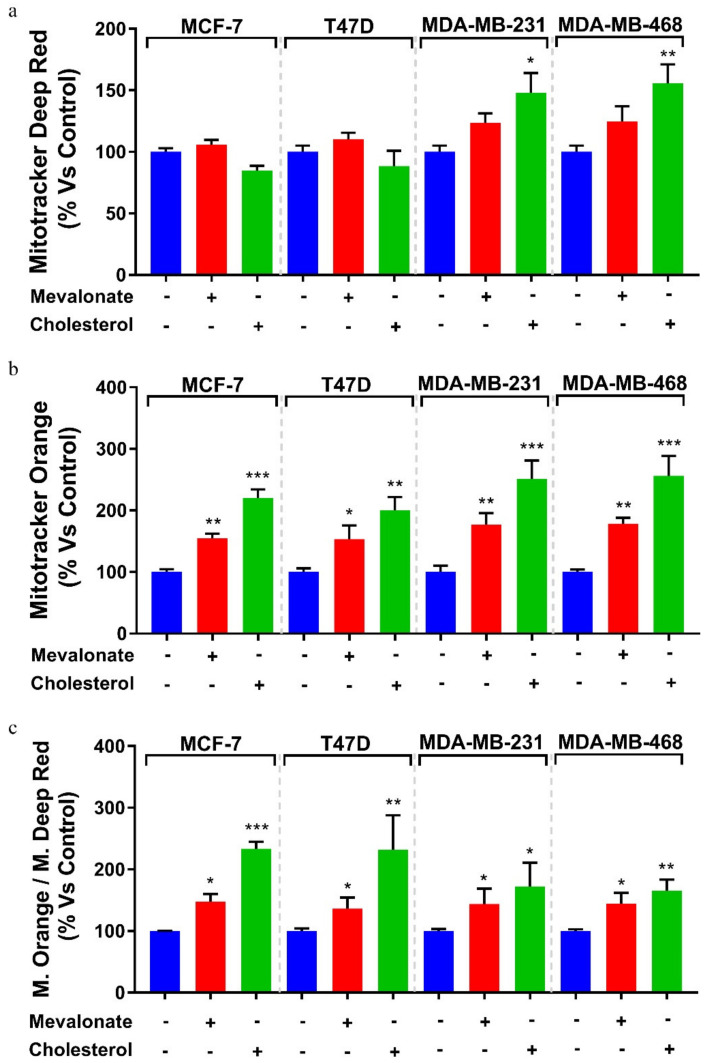
Mevalonate and cholesterol induce mitochondrial activity. Mitochondrial mass (**a**) and potential (**b**) after 5 days of 1 mM mevalonate or 10 µM cholesterol treatment were assessed using MitoTracker Deep Red FM and MitoTracker Orange CM-H2TMRos probes, respectively, in MCF7, T47D, MDA-MB-231, and MDA-MB-468. (**c**) Ratio between potential and mitochondrial mass. Values are expressed as the mean ± SD of three independent experiments. * *p*-value < 0.05; ** *p*-value < 0.01; *** *p*-value < 0.001.

Based on the immunoblot results in which we demonstrated high expression levels of ERRα and of PGC1α, as well as high expression levels of ERbB2/HER2, representing a tyrosine kinase receptor highly implicated in the control of various metabolic processes, we analyzed the metabolic profile of all breast cancer cell lines after treatment with mevalonate or cholesterol for 48 h, by using a Seahorse XFe96 analyzer. The mitochondrial function was assessed by performing a Mito Stress test. Monitoring the oxygen consumption rate (OCR) after sequential injections of oligomycin, FCCP, and an antimycin/rotenone mix allowed us to evaluate the mitochondrial function parameters, such as basal respiration and maximal respiration. Mevalonate and cholesterol treatment induced a marked increase of all the above-mentioned mitochondrial respiration parameters (Figure 6). Additionally, we found increased ATP production, which was assessed using a CellTiter-glo assay. Glycolytic function was detected by monitoring the extracellular acidification rate (ECAR); sequential injections of glucose, oligomycin, and 2-deoxy-D-glucose (2-DG) allowed us to evaluate the glycolytic function parameters, such as the glycolytic capacity and glycolytic reserve. Interestingly, MCF-7, T47D, and MDA-MB-231 cells, treated with mevalonate or cholesterol for 48 h, showed an increase in glycolysis and in all glycolytic function parameters (Figure 7), while the ECAR profile and other glycolytic function parameters were not affected in MDA-MB-468 cells, which may have been due to the higher basal glycolytic activity (Figure 6).

To confirm the OCR results, the mitochondrial mass, mitochondrial potential, and their ratio, which is an index of the mitochondrial function, were evaluated after 5 days of treatment with cholesterol or mevalonate in all examined breast cancer lines.

**Figure 8:**
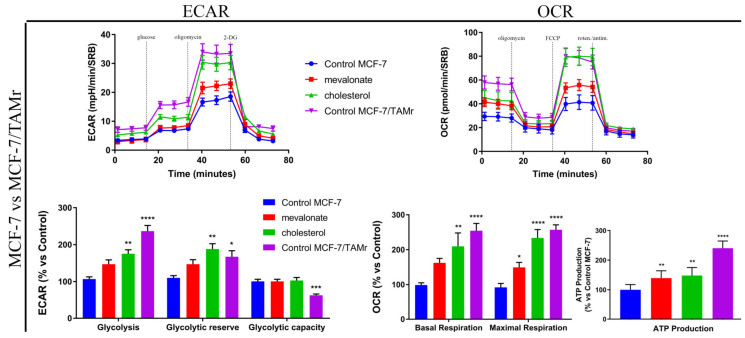
Mevalonate and cholesterol increase the metabolic functions in MCF-7, inducing the same metabolic profile of MCF-7/TAMr. A Seahorse XFe96 analyzer was employed to analyze the mitochondrial respiration and glycolytic function in MCF-7 cells treated with mevalonate or cholesterol for 48 h (the seahorse analysis in Figure 8 is the same as the one shown in Figure 6 for MCF7 cells) by monitoring the oxygen consumption rate (OCR) and extracellular acidification rate (ECAR), respectively, compared with MCF-7/TAMr. Histograms of mitochondrial respiration parameters and histograms of glycolytic function parameters are also shown and compared with untreated MCF-7/TAMr. Values are expressed as the mean ± SEM of five biological replicates of three independent experiments. * *p*-value < 0.05; ** *p*-value < 0.01; *** *p*-value < 0.001; **** *p*-value < 0.0001.

We observed an increased mitochondrial mass in all studied triple negative breast cancer cell lines; conversely, no change was found in ERα+ cell lines (Figure 7). However, in all cell lines, both the mitochondrial potential and the ratio of mitochondrial membrane potential versus mitochondrial mass increased (Figure 7), confirming the OCR results obtained by Seahorse analysis. Overall, our results highlight the ability of cholesterol and mevalonate to increase the energy metabolism of breast cancer cells, upregulating glycolysis and oxidative phosphorylation, which are the two main pathways of cell metabolism. Moreover, the metabolic functions of MCF-7/TAMr and MCF-7 cells treated with mevalonate or cholesterol were compared (Figure 8), highlighting the ability of the two metabolites to induce, in MCF-7, the same metabolic profile of ERα+ cells insensible to endocrine therapy.

**Figure 9:**
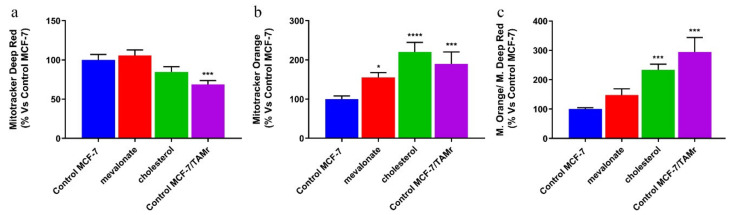
MCF-7 cells treated with mevalonate or cholesterol showed mitochondrial activity comparable to that of the MCF-7/TAMr cell line. Mitochondrial mass (**a**) and potential (**b**) after 5 days of 1 mM mevalonate or 10 µM cholesterol treatment were assessed using MitoTracker Deep Red FM and MitoTracker Orange CM-H2TMRos probes, respectively, in MCF7 (mitochondrial mass and mitochondrial potential analysis shown in Figure 8 is the same as the one shown in Figure 7 for MCF7 cells) versus MCF-7/TAMr. (**c**) Ratio between potential and mitochondrial mass. Values are expressed as the mean ± SD of three independent experiments. * *p*-value < 0.05; *** *p*-value < 0.001; **** *p*-value < 0.0001.

In addition, we found that both the mitochondrial potential and the ratio of mitochondrial membrane potential versus mitochondrial mass of MCF-7 cells were also comparable to those of MCF-7/TAMr (Figure 9).

**Figure 10:**
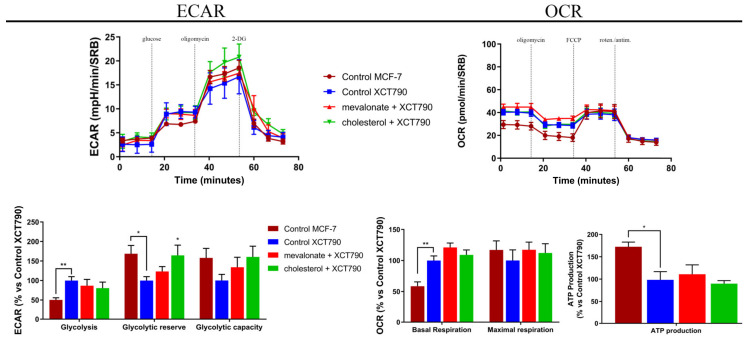
MCF-7 cells co-treated with mevalonate or cholesterol and XCT790 showed no relevant metabolic effects on the MCF-7 cell line. A Seahorse XFe96 analyzer was employed to analyze the mitochondrial respiration and glycolytic function in MCF-7cells treated with the vehicle alone or co-treated with 5 µM XCT790 and mevalonate or cholesterol (1 mM and 10 µM, respectively) for 48 h by monitoring the oxygen consumption rate (OCR) and extracellular acidification rate (ECAR), respectively. Histograms of mitochondrial respiration parameters and histograms of glycolytic function parameters are also shown. Values represent the mean ± SEM of five biological replicates of three independent experiments. * *p*-value < 0.05; ** *p*-value < 0.01.

The strong energy boost detected in all examined cell lines in response to treatment with cholesterol or mevalonate, its metabolic precursor, prompted us to hypothesize that it could be due to ERRα pathway activation and increased expression levels of ERbB2/HER2, which are proteins involved in metabolism regulation and modulated by ERRα. The implication of the ERRα pathway in the observed metabolic effects was confirmed by seahorse analysis performed in MCF-7 co-treated with cholesterol or mevalonate and XCT790, which is a selective inverse agonist ligand of ERRα (Figure 10). In these conditions, no metabolic boost induced by the two metabolites was observed.

### 3.5. Mevalonate and Cholesterol Increase Breast Cancer Stem Cell Propagation

**Figure 11:**
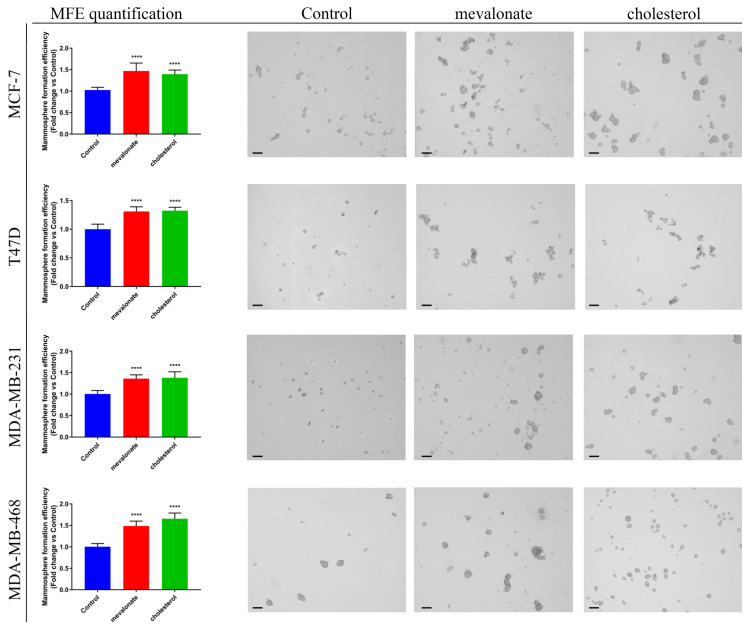
Cholesterol and mevalonate promote cancer stem-like cell (CSC) propagation. A mammosphere formation efficiency assay was performed on breast cancer cells MCF7, T47D, MDA-MB-231, and MDA-MB-468 after treatment for 5 days with 1 mM mevalonate or 10 µM cholesterol. Mammospheres were grown in no-attachment conditions and counted using an Olympus BX41 (4× magnification) microscope. 3D-spheres > 50 μm were counted using an eye piece (“graticule”), and the percentage of cells plated that formed spheres was calculated and referred to as the percent mammosphere formation efficiency (MFE). Results are expressed as the mean ± SD of three independent experiments. Scale bars are 125 µm. **** *p*-value < 0.0001.

Since cholesterol and its biosynthetic precursor, mevalonate, were revealed to strongly increase the cancer cell mitochondrial function, next, we investigated whether they may affect cancer stem cell propagation. CSCs are a sub-population of tumor cells responsible for therapy resistance and tumor recurrence [49], and mitochondrial function is required for their anchorage-independent survival and propagation [30]. To this end, we monitored the mammosphere formation efficiency (MFE) after treatment for 5 days with mevalonate and cholesterol. We found that mevalonate and cholesterol treatment significantly increased breast cancer stem cell propagation (Figure 11), suggesting that the activation of ERRα and related pathways, by cholesterol and mevalonate, may work together to determine an aggressive and likely resistant breast cancer phenotype.

### 3.6. Increase of Lipid Droplet Formation in Mevalonate- and Cholesterol-Treated Breast Cancer Cells

**Figure 12:**
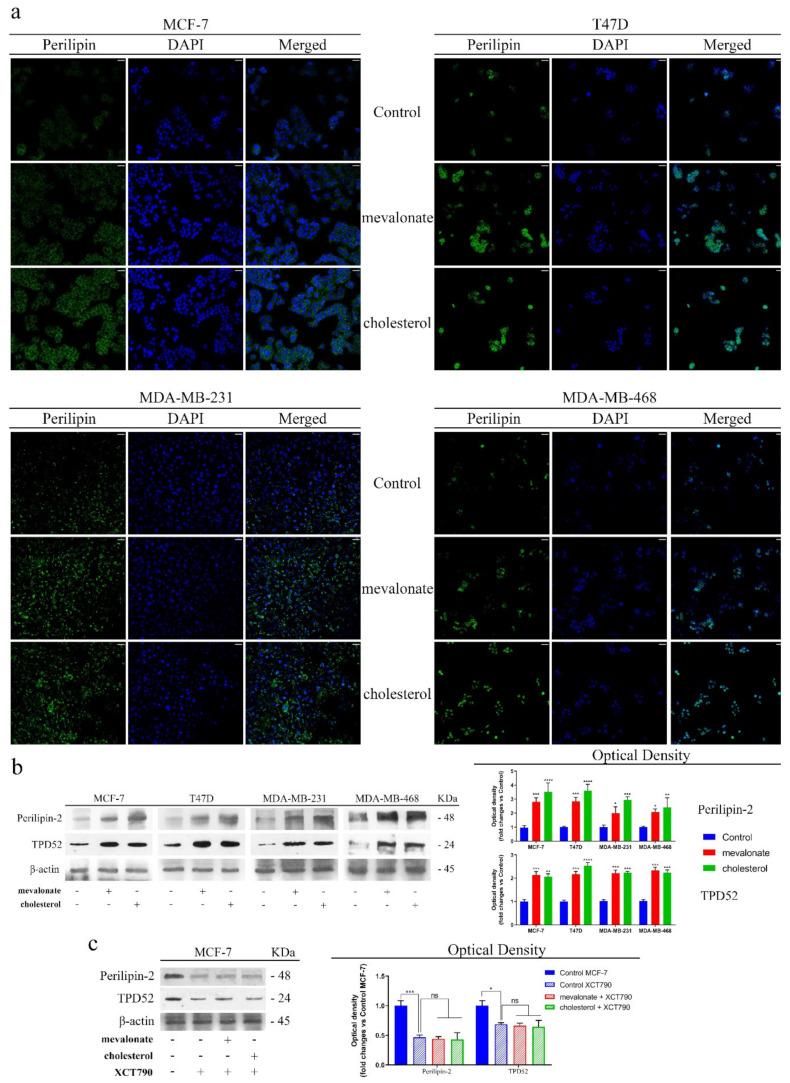
Cholesterol and mevalonate promote perilipin-2 expression through ERRα activation. (**a**) Immunofluorescence and (**b**) immunoblot analysis of perilipin-2 and TPD52 were performed on breast cancer cells MCF7, T47D, MDA-MB-231, and MDA-MB-468 after treatment for 48 h with 1 mM mevalonate or 10 µM cholesterol. (**c**) Immunoblot analysis on MCF-7 of perilipin-2 and TPD52 after co-treatment with 5 µM XCT790, 1 mM mevalonate, or 10 µM cholesterol for 2 days. Pictures were taken at 20× magnification. Scale bars are 25 µm. Results are expressed as the mean ± SD of three independent experiments. * *p*-value < 0.05; ** *p*-value < 0.01; *** *p*-value < 0.001; **** *p*-value < 0.0001.

We then evaluated lipid droplet (LDs) formation, which is an additional hallmark that could play an important role in contributing to the aggressive phenotype and resistance onset. Lipid droplets are storage organelles present in low numbers in most cell types, while increased numbers can occur in cancer cells. In recent years, much focus has been given to the lipid droplets and in particular, to the relationship between lipid droplets and resistance to chemotherapy drugs by tumor cells [50]. Based on the involvement of LDs in aggressive cancer phenotypes, we evaluated perilipin-2, which is a protein constituent of LDs, after treatment with mevalonate or cholesterol, by immunofluorescence and immunoblot analyses. The results (Figure 12) showed a marked increase in perilipin-2 expression levels in treated cells. Interestingly, the immunofluorescence study revealed differences in the perilipin-2 content, which increased in parallel with the aggressiveness of the analyzed breast cancer cells. Moreover, mevalonate and cholesterol treatment resulted in higher expression levels of a protein closely connected to ERbB2 and LD formation,—tumor protein D52 (TPD52)—representing an oncogene whose overexpression has been associated with poor outcomes in breast malignancies [51]. Furthermore, the higher perilipin-2 and TPD52 expression levels induced by cholesterol or mevalonate treatment were dependent on ERRα activity, since XCT790 treatment abolished them (Figure 12 and Figure S2).

**Figure 13:**
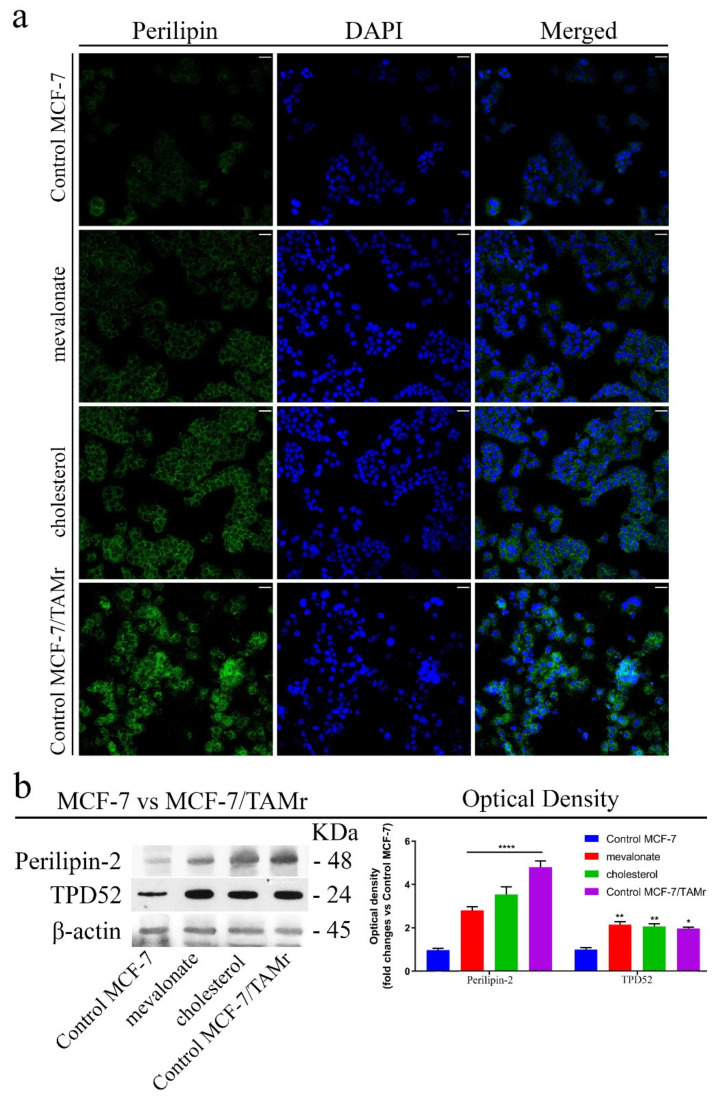
In MCF-7 cells, mevalonate and cholesterol treatment induces perilipin expression levels comparable to those of MCF-7/TAMr cells. (**a**) Immunofluorescence of perilipin-2 and (**b**) immunoblot analysis of perilipin-2 and TPD52 after treatment with 1 mM mevalonate or 10 µM cholesterol for 2 days. The immunofluorescence shown in (**a**) and the western blot shown in (**b**) are the same as the those shown in Figure 12 for MCF-7 cells. Pictures were taken at 20× magnification. Scale bars are 25 µm. Results are expressed as the mean ± SD of three independent experiments. * *p*-value < 0.05; ** *p*-value < 0.01; **** *p*-value < 0.0001.

Finally, we compared perilipin-2 and TPD52 expression levels in MCF-7/TAMr versus MCF-7 cells treated with mevalonate or cholesterol (Figure 13) and obtained comparable results.

### 3.7. Mevalonate and Cholesterol Induce Chemotherapy Drug Resistance

**Figure 14:**
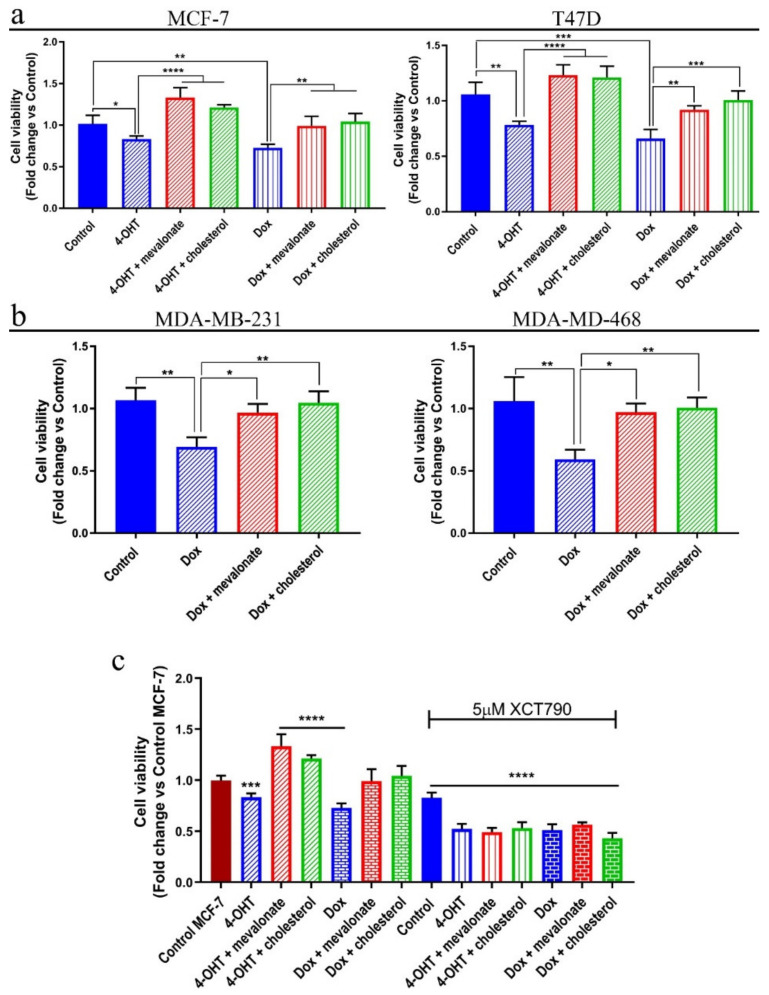
Cholesterol and mevalonate promote resistance to common chemotherapy drugs through ERRα activation. Breast cancer cells (**a**) MCF7 and T47D and (**b**) MDA-MB-231 and MDA-MB-468 were treated with 1 mM mevalonate or 10 µM cholesterol in the presence of 5 µM 4-OH-tamoxifen (4-OHT) or 1 µM doxorubicin (Dox) for 48 h. (**c**) MCF-7 cells were treated with 1 mM mevalonate or 10 µM cholesterol in the presence of 4-OH-tamoxifen (4-OHT) or doxorubicin (Dox) and 5 µM XCT790 for 48 h. Cell viability was assessed using SRB. The results obtained from the SRB assay were related to their own control. Values are indicated as the mean ± SD of three independent experiments. * *p*-value < 0.05; ** *p*-value < 0.01; *** *p*-value < 0.001; **** *p*-value < 0.0001.

Since we established that cholesterol and mevalonate, through ERRα, activate pathways involved in tumor progression and drug resistance, we asked whether the two metabolites were really able to confer resistance to drugs currently used in clinical practice, such as tamoxifen and doxorubicin. To this end, using an SRB assay, we evaluated the cell viability after 48 h of treatment with mevalonate or cholesterol in the presence or absence of 4-OH-tamoxifen or doxorubicin. We compared the results of each breast cancer cell line versus each untreated control. As shown in Figure 14, all of the examined breast cancer cell lines exhibited an increase in the resistance to the 4-OH-tamoxifen or doxorubicin, confirming that cholesterol and mevalonate induce chemotherapy resistance in breast cancer cells. The observed doxorubicin resistance finds validation in a previous report, which linked the antitumor effect of doxorubicin to its inhibitory effect on the HMGCR function [52]. Moreover, to confirm the close correlation between drug resistance induced by cholesterol and mevalonate through both ERRα and related pathway activation, we performed the same experiment in the presence of XCT790. We used the MCF-7 cell line (representative of ER+ cells) (Figure 14) and MDA-MB-231 cell line (representative of triple negative cells) (Figure S2). The results evidenced that XCT790 is able to revert the cholesterol and mevalonate effect to induce tamoxifen and doxorubicin resistance, confirming that the resistance induction is mediated by ERRα and related pathway activation.

### 3.8. High Levels of ERRα are Associated with a Poor Prognosis in Breast Cancer Patients

**Figure 15:**
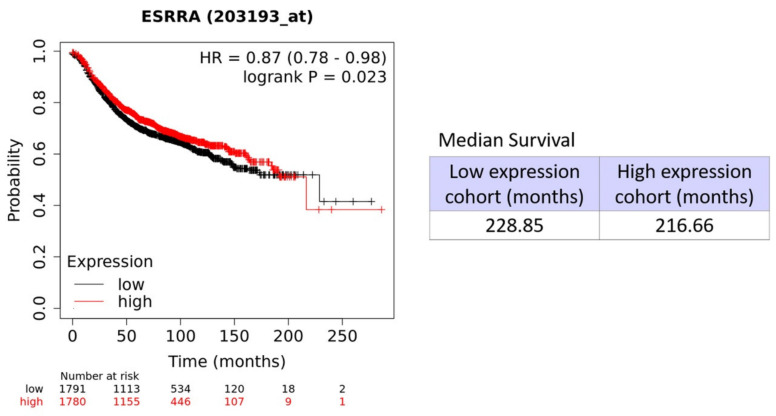
Prognostic value of ERRα in human breast cancer subtypes. To assess the clinical relevance of ERRα, we determined the ERRα mRNA transcript levels in human breast cancer patient cohorts with long-term follow-up data. Note that high mRNA levels of ERRα show an association with reduced relapse-free survival (RFS), i.e., a higher tumor recurrence.

Based on the results above, in which we demonstrated that the activation of ERRα by cholesterol or mevalonate was able to confer resistance to chemotherapy, as well as to promote breast cancer aggressiveness, we assessed the prognostic value of ERRα in patients with breast cancer (all breast cancer subtypes). We used publicly available transcriptional profiling data from tumors of breast cancer patients (Gene Probe 203193_at). The hazard-ratio for ERRα was 0.87 (N = 3571 patients) (Figure 15 and Table 1) for relapse-free survival (RFS), highlighting that the expression of ERRα is a negative prognostic marker in patients with breast cancer.

## 4. Discussion

Recurrence, metastasis, and therapy resistance remain unmet clinical needs that require new therapeutic strategies [53]. In this study, we focused our attention on cholesterol and its biosynthetic precursor, mevalonate, as two biomolecules that can play key roles during the progression of breast cancer and resistance to common therapy. It is well-known that the cholesterol level is a risk factor of breast cancer [54], promotes resistance to tamoxifen in ER+ breast cancer cells, and is also associated with ERRα pathway activation. Considering this, we analyzed the metabolic and biological changes that cholesterol and mevalonate are able to induce in breast cancer cells. We used a panel of breast cancer cells that reflect the biological diversity of the different breast cancer subtypes, such as MCF-7, T47D, MDA-MB-231, and MDA-MB-468 cells. Our results showed that mevalonate and cholesterol are able to activate the ERRα pathway, regardless of the subtype, promoting the expression of two main proteins: PGC-1α, an important co-activator of ERRα signaling, acting on mitochondrial biogenesis [55] and ERbB2/HER2, which is a tyrosine kinase receptor closely dependent on the ERRα pathway and implicated in cancer maintenance and progression [45,46]. These two pathway are strictly related as they drive the metabolism of breast cancer cells. Moreover, we found that cholesterol or mevalonate treatment induces a higher expression of TPD52, which is the tumor protein D52 dependent on ERbB2/HER2 [56]. Surprisingly, the expression level of these proteins in MCF-7 cells treated with cholesterol or mevalonate was comparable to that observed in MCF-7/TAMr cells resistant to tamoxifen, confirming that cholesterol or mevalonate, acting on the ERRα pathway, may induce aggressive and resistant phenotypes. The activation of the ERRα pathway by the treatment was also confirmed by using XCT790, which is a selective ERRα inhibitor. In order to study the behavior of cholesterol or mevalonate during cancer progression, we focused the attention on the main cancer features, such as cell proliferation and migration. Abnormal cellular proliferation due to a dysregulated cell cycle is one of the hallmarks of cancer [57]. Using cytofluorimetric analysis, we demonstrated that mevalonate and cholesterol promote cell cycle progression, increasing the G2/M phase, with the latter being an immediate consequence of the previously observed pathway activation. Importantly, we found that cholesterol and mevalonate treatment can enhance cell motility, highlighting that the two metabolites are related to breast cancer aggressiveness.

Metabolic reprogramming is a hallmark of cancer; altered metabolic pathways are indeed prognostic markers of disease and very important pharmacological targets for cancer therapy [58,59]. Depending on the availability of nutrients, some cells within the tumor are predominantly glycolytic, while others have a phenotype dependent on oxidative phosphorylation [60,61]. Therefore, we evaluated the energy profile of cells treated with mevalonate or cholesterol and the results indicate the ability of these metabolites to increase the two main cellular energy pathways: Glycolysis and oxidative phosphorylation. These results were confirmed by cytofluorimetric analysis performed in order to evaluate the mitochondrial mass, mitochondrial potential, and their ratio, known as the index of mitochondrial function. Our findings showed that the mitochondrial membrane potential increased in all tested breast cancer cells, while the mitochondrial mass only increased in triple negative cell lines. Importantly, the ratio of mitochondrial membrane potential versus mitochondrial mass increased in all breast cancer cells examined. We demonstrated that this is completely related to the effect of cholesterol and mevalonate on ERRα and the signaling pathways that it triggers. In particular, PGC-1α and ERbB2/HER2, involved in mitochondria biogenesis and the stimulation of oxidative metabolism, respectively, were affected. In this context, we proved that the effect of the two metabolites was reverted in MCF-7 cells treated with mevalonate or cholesterol in the presence of XCT790—the ERRα inhibitor.

In addition to that, we showed that mevalonate and cholesterol increase the growth and propagation of CSCs, representing the subpopulation of tumor cells mainly responsible for cancer resistance, recurrence, and metastasis. CSCs have a high metabolic flexibility that allows them to switch between the glycolytic and phosphorylative pathways, according to the energy needs of the malignant cell [62,63,64]. Indeed, our results indicate that mevalonate and cholesterol promote CSC formation and propagation, increasing their energy metabolism and promoting the metabolic flexibility responsible for their aggressiveness.

In order to better define the contribution of mevalonate and cholesterol in resistance to anti-cancer therapy used in clinical practice, we focused our attention on lipid droplets (LDs), which have increasingly become a major topic of research during last years. Recent studies have explained that LDs are not passive cytoplasmic structures, but interact with different organelles to modulate several cellular processes [65]. Increased LD formation in cancer cells can be correlated with anti-cancer therapy resistance. Resistance to anti-cancer drugs is a persistent problem in treating tumors and varies tremendously among different cancer types, but lipid metabolism can be a common crucial contributor [66,67]. Interestingly, we demonstrated that the activation of ERRα, by cholesterol or mevalonate treatment, noticeably increased the perilipin expression level. Our results are in agreement with the high levels of TPD52 protein detected in MCF-7 cells after mevalonate or cholesterol treatment. Both proteins are related to lipid droplet formation [68]. Indeed, our findings established that treatment with cholesterol or mevalonate is able to increase the amount of perilipin-2 in all breast cancer cell lines examined, determining a further feature that could lead to greater aggressiveness and resistance to pharmacological treatments currently used in clinical practice, induced by mevalonate and cholesterol treatment.

These data, altogether, suggest that cholesterol or mevalonate treatment, through the activation of ERRα and related pathways, can significantly reduce the antiproliferative and cytotoxic effects of clinically used tamoxifen and doxorubicin. These metabolic and biological changes, induced by the treatments, are used by the breast cancer cell to develop a resistant and aggressive phenotype. Overall, our outcomes highlight how high cholesterol levels are able to trigger, by themselves, aggressiveness and tumor resistance. Moreover, the results obtained in this study showed very similar effects induced by the treatment with mevalonate, which can be traced back to its conversion to cholesterol. Furthermore, Kaplan–Meier analyses, performed on patients with any breast cancer subtype, confirmed that a poor prognosis is associated with high levels of ERRα [69,70], opening new scenarios in the treatment of breast cancer in light of the strong connection between mevalonate/cholesterol and activation of the ERRa pathway.

## Figures and Tables

**Table 1:** Prognostic value of ERRα in human breast cancer subtypes: Tumor recurrences (RFS).

Symbol	Gene Probe	HR (Hazard Ratio)	*p*-Value (Log Rank Test)
All Breast Cancer Subtypes *n* = 3571			
ERRα/ESRRA	203193_at	0.87	0.023

## Supplementary Materials

The following are available online at https://www.mdpi.com/2073-4409/9/8/1819/s1, Figure S1. Immunoblot analysis of ERRα and PGC1α in MCF-7 cells under starvation conditions after treatment with 1 mM mevalonate or 10 µM cholesterol for 2 days, Figure S2. Co-treatment with XCT790 and mevalonate or cholesterol reduces ERRα expression levels, main correlated proteins and drug resistance in MDA-MB-231, Figure S3. Cell growth analysis, assessed by SRB assay, of MCF-7, T47D, MDA-MB-231, MDA-MB-468 cells treated for 3 or 5 days with EtOH (Control) or 1 mM mevalonate or 10 µM cholesterol, Figure S4. Wound-healing scratch assay in MCF7 cells treated for 24 h with EtOH (Control) or 1 mM mevalonate or 10 µM cholesterol, Figure S5. Wound-healing scratch assay in T47D cells treated for 24 h with EtOH (Control) or 1 mM mevalonate or 10 µM cholesterol, Figure S6. Wound-healing scratch assay in MDA-MB-231 cells treated for 24 h with EtOH (Control) or 1 mM mevalonate or 10 µM cholesterol, Figure S7. Wound-healing scratch assay in MDA-MB-468 cells treated for 24 h with EtOH (Control) or 1 mM mevalonate or 10 µM cholesterol.
