# Supplementary material for: Cholesterol and Mevalonate: Two Metabolites Involved in Breast Cancer Progression and Drug Resistance through the ERRα Pathway
Source: Cells. 2020 Jul 31;9(8):1819. doi: 10.3390/cells9081819 (PMC7465765; doi:10.3390/cells9081819)
Supplement: Supplementary file 1 [file cells-09-01819-s001.pdf]

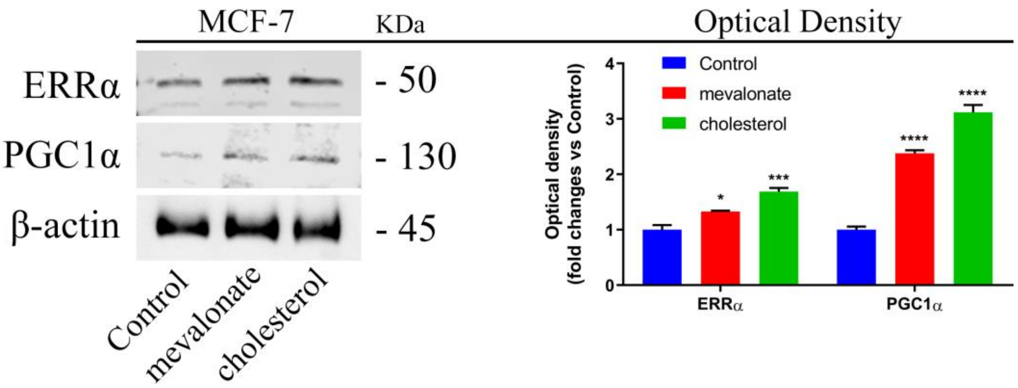

Figure S1. Immunoblot analysis of ERRα and PGC1α in MCF-7 cells under starvation conditions after treatment with 1 mM mevalonate or 10 μM cholesterol for 2 days. Results obtained from densitometry were related to control and represent the mean ± SD of three independent experiments. * p value < 0.05; *** p value < 0.001; **** p value < 0.0001.

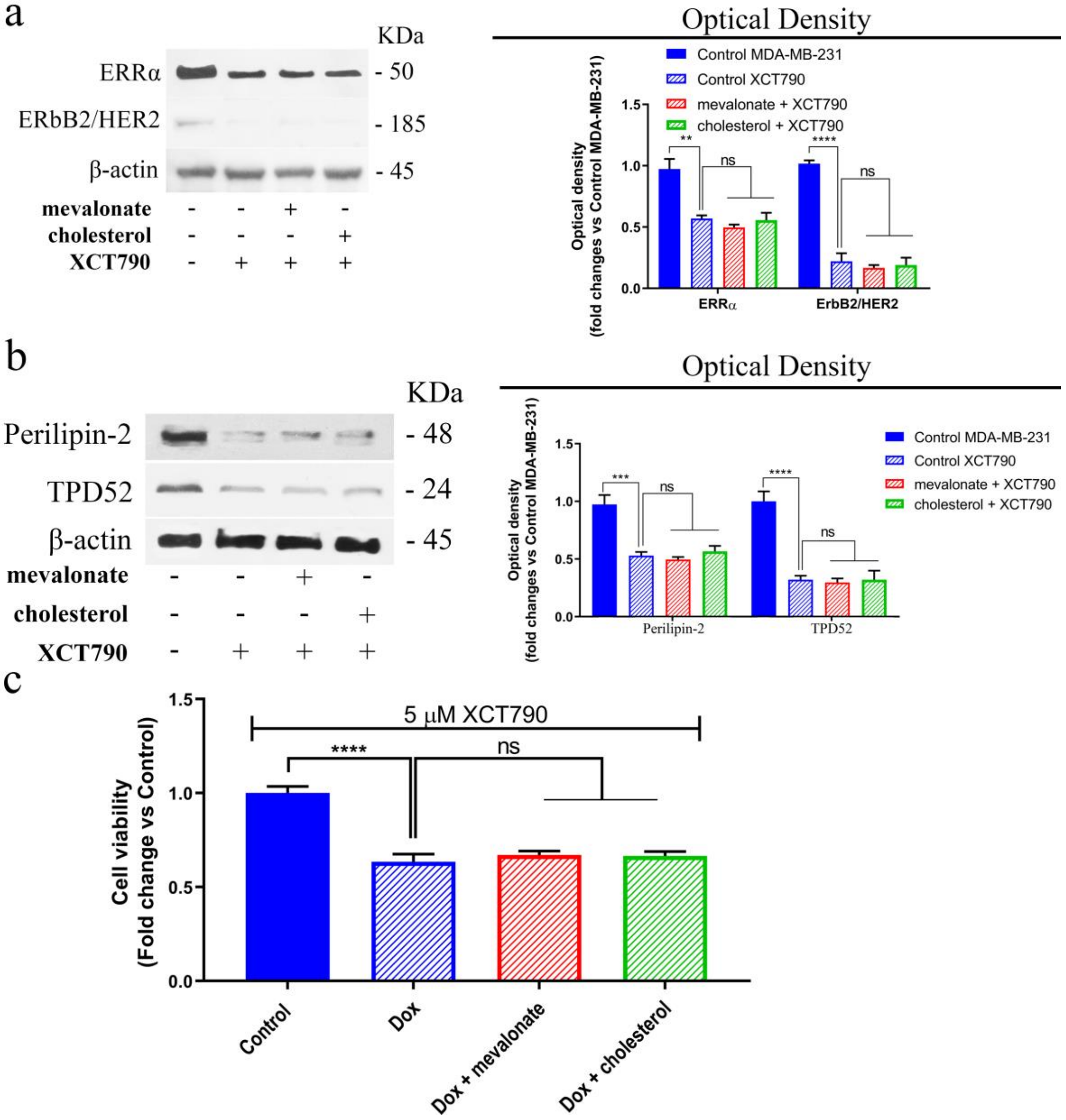

Figure S2. Co-treatment with XCT790 and mevalonate or cholesterol reduces ERRα expression levels, main correlated proteins and drug resistance in MDA-MB-231. (a) Immunoblot analysis of ERRα and

ERbB2/HER2 after co-treatment with 5 μM XCT790, 1 mM mevalonate or 10 μM cholesterol for 2 days. Results obtained from densitometry were related to their own control. (b) Immunoblot analysis of perilipin-2 and TPD52 after co-treatment with 5 μM XCT790, 1 mM mevalonate or 10 μM cholesterol for 2 days. Results obtained from densitometry were related to their own control. (c) MDA-MB-231 cells were treated with 1 mM mevalonate or 10 μM cholesterol in presence of doxorubicin (Dox) and 5 μM XCT790 for 48 h. Cell viability was assessed using SRB. Results obtained from SRB assay were related to their own control. Values represent the mean ± SD of three independent experiments. ** p value < 0.01; *** p value < 0.001; **** p value < 0.0001.

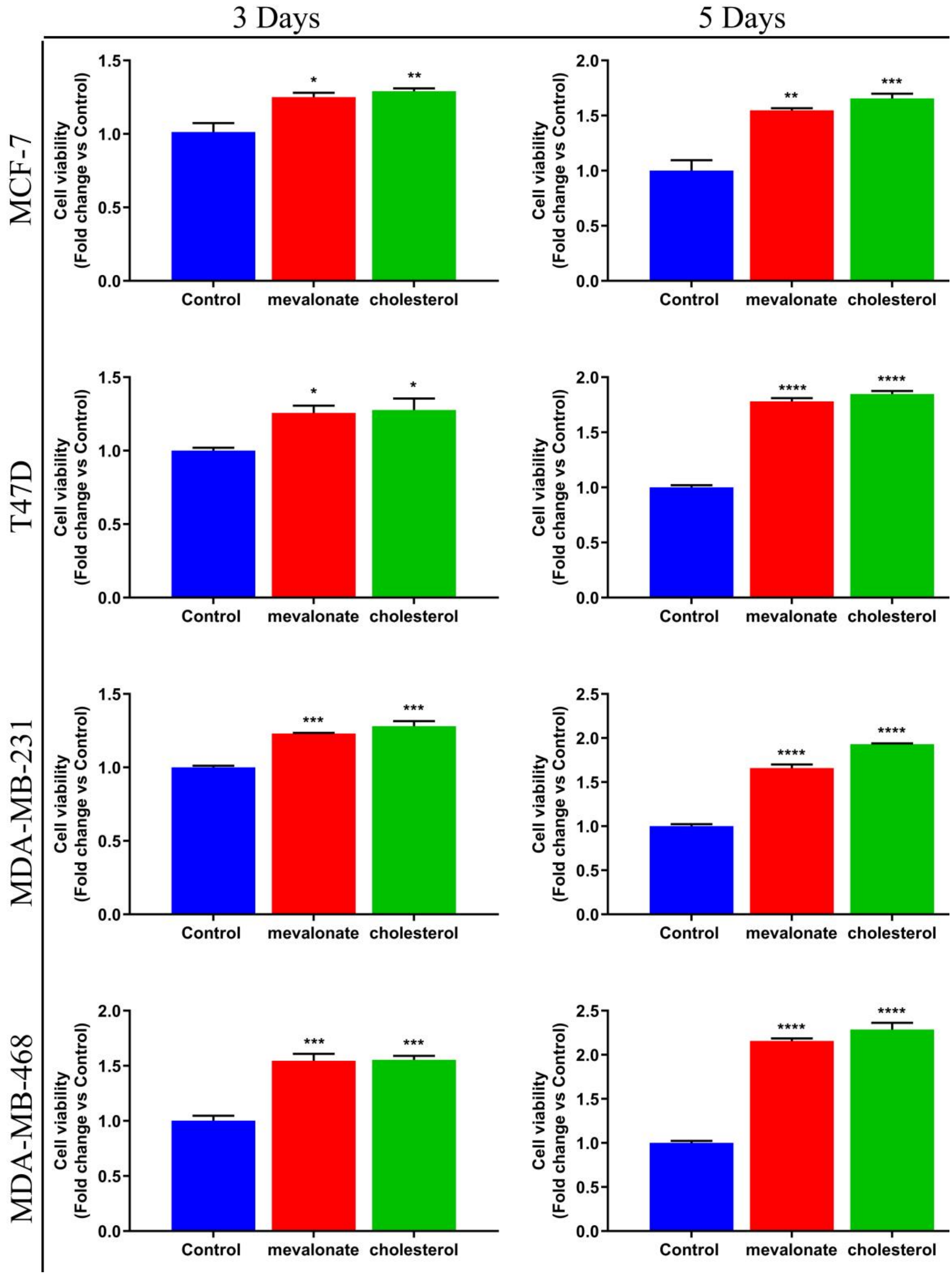

Figure S3. Cell growth analysis, assessed by SRB assay, of MCF-7, T47D, MDA-MB-231, MDA-MB-468 cells treated for 3 or 5 days with EtOH (Control) or 1 mM mevalonate or 10 μM cholesterol. * *p* value <0.05; ** *p* value < 0.01; *** *p* value < 0.001; **** *p* value < 0.0001.

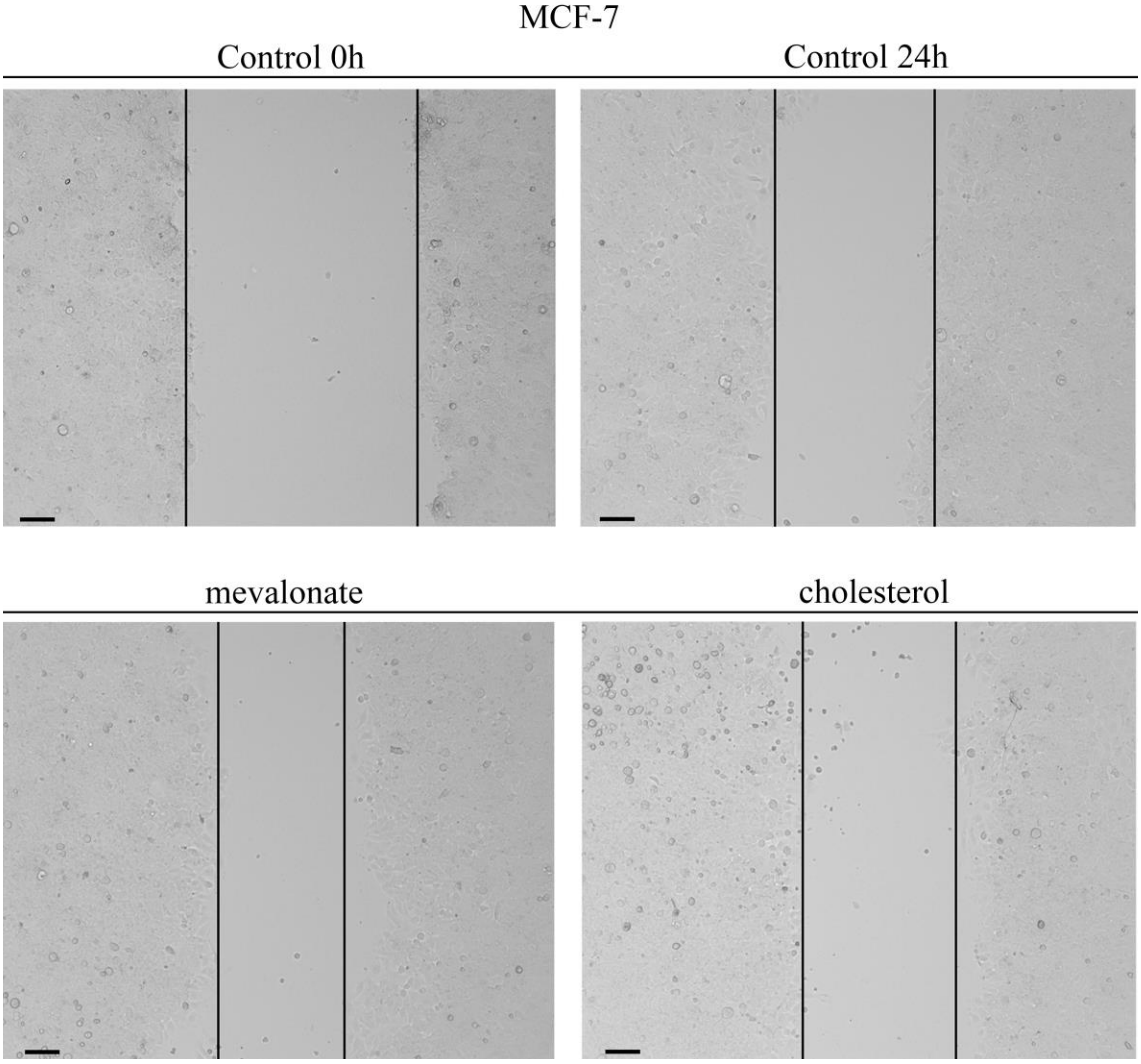

Figure S4. Wound-healing scratch assay in MCF7 cells treated for 24 h with EtOH (Control) or 1 mM mevalonate or 10 μM cholesterol. Pictures were taken at 10× magnification using EVOS FL Auto 2 microscope. Scale bars 50 μm.

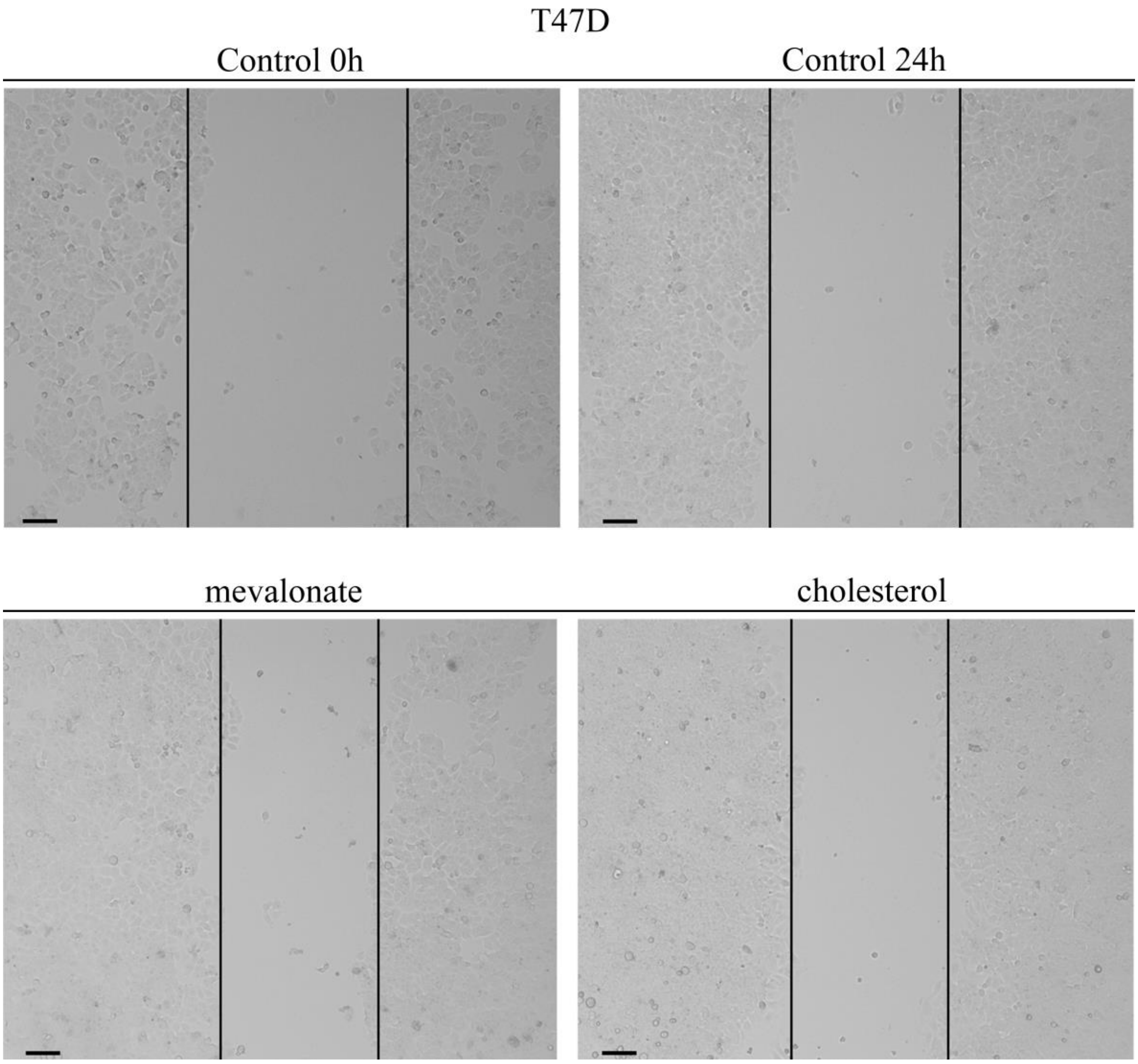

Figure S5. Wound-healing scratch assay in T47D cells treated for 24 h with EtOH (Control) or 1 mM mevalonate or 10 μM cholesterol. Pictures were taken at 10× magnification using EVOS FL Auto 2 microscope. Scale bars 50 μm.

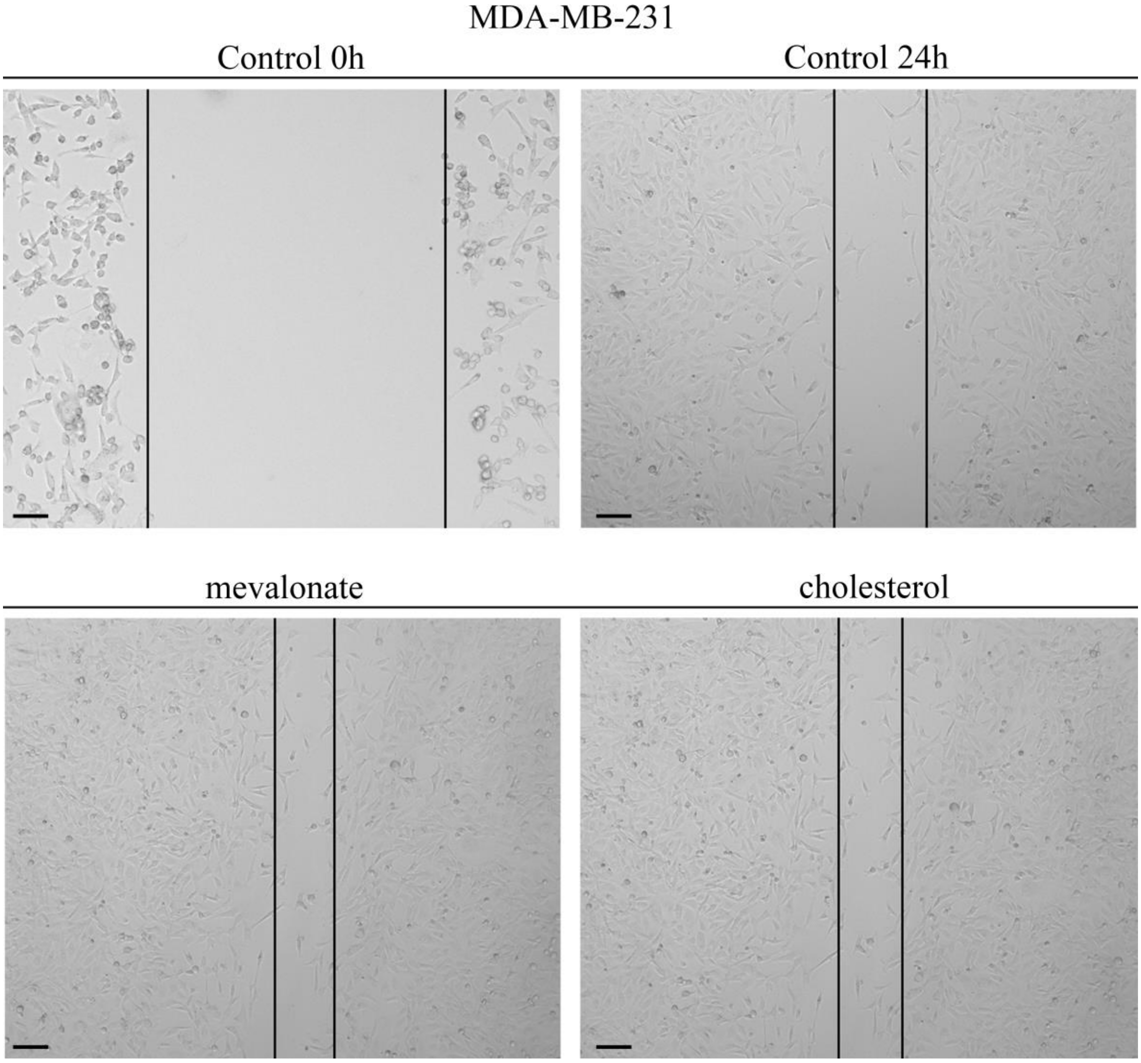

Figure S6. Wound-healing scratch assay in MDA-MB-231 cells treated for 24 h with EtOH (Control) or 1 mM mevalonate or 10 μM cholesterol. Pictures were taken at 10× magnification using EVOS FL Auto 2 microscope. Scale bars 50 μm.

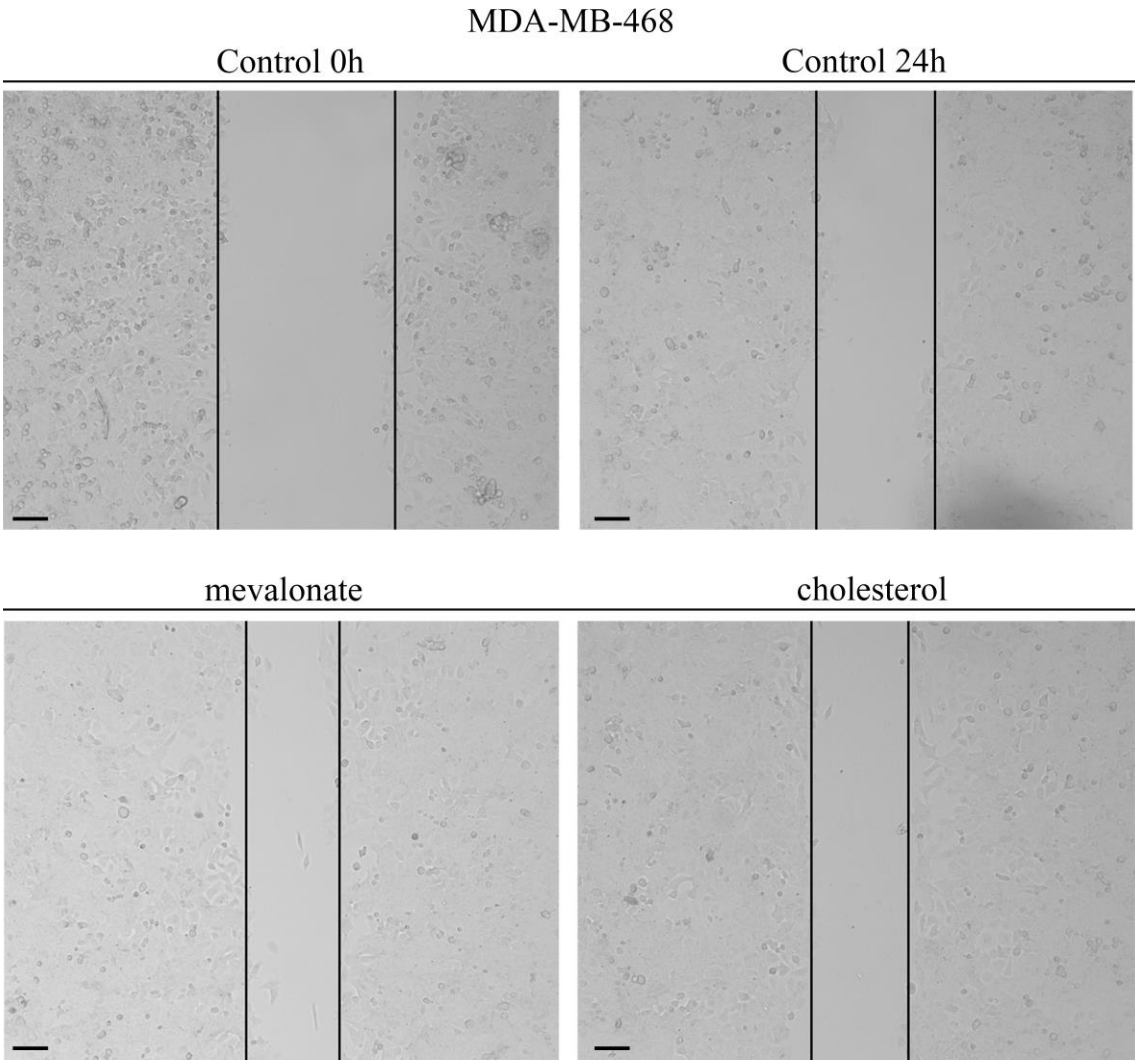

Figure S7. Wound-healing scratch assay in MDA-MB-468 cells treated for 24 h with EtOH (Control) or 1 mM mevalonate or 10 μM cholesterol. Pictures were taken at 10× magnification using EVOS FL Auto 2 microscope. Scale bars 50 μm.
